# Identification of a Testis-Enriched Heat Shock Protein and Fourteen Members of *Hsp70* Family in the Swamp Eel

**DOI:** 10.1371/journal.pone.0065269

**Published:** 2013-06-04

**Authors:** Yan He, Majing Luo, Minhan Yi, Yue Sheng, Yibin Cheng, Rongjia Zhou, Hanhua Cheng

**Affiliations:** 1 College of Fisheries, Huazhong Agricultural University, Wuhan, P.R. China; 2 Department of Genetics, College of Life Sciences, Wuhan University, Wuhan, P.R. China; Nanjing Agricultural University, China

## Abstract

**Background:**

Gonad differentiation is one of the most important developmental events in vertebrates. Some heat shock proteins are associated with gonad development. Heat shock protein 70 (Hsp70) in the teleost fish and its roles in sex differentiation are poorly understood.

**Methods and Findings:**

We have identified a testis-enriched heat shock protein Hspa8b2 in the swamp eel using Western blot analysis and Mass Spectrometry (MS). Fourteen *Hsp70* family genes were further identified in this species based on transcriptome information. The phylogenetic tree of *Hsp70* family was constructed using the Maximum Likelihood method and their expression patterns in the swamp eel gonads were analyzed by reverse transcription-polymerase chain reaction (RT-PCR).

**Conclusion:**

There are fourteen gene members in the *Hsp70* family in the swamp eel genome. *Hsp70* family, particularly *Hspa8*, has expanded in the species. One of the family members *Hspa8b2* is predominantly expressed in testis of the swamp eel.

## Introduction

Gonad differentiation is one of the most important developmental events in vertebrates, and the underlying molecular mechanism is always a debate focus. A number of key genes involved in sex determination have been identified in mammals, such as *Sry* (sex-determining region on Y chromosome), which encodes a DNA-binding protein that acts dominantly to trigger differentiation of testes from undifferential gonads [1], [2]. *Sox9* can induce testis development in the XX transgenic mice (*Mus musculus*), a condition of complete absence of *Sry* [3], [4] and it may be a direct downstream target of *Sry* to initiate the male development. However, *Sox9* is dispensable during subsequent embryonic and postnatal testis development, only leads to late-onset sterility at about 5 months [5], [6]. *Sox8* may play a role in maintenance of the integrity of the basal lamina [7]. Together, sex determination in mammals is regulated by two groups of factors: *Sox* (*Sry, Sox8, Sox9* and *Sox10*) and the *Rspo1/Wnt/beta-catenin* [8], [9], [10], [11]. In addition, insulin and Igf1 receptors are essential for testis determination and/or differentiation in mice [12], [13], and *Dmrt1* is evolutionarily conserved and play a prominent role in regulation of testicular differentiation and gametogenesis in vertebrates [6], [14]. In fish, *Dmy/Dmrt1Y,* which is a duplicated copy of *Dmrt1* on the Y chromosome, is required for male sex determination in the teleost fish medaka (*Oryzias latipes*) [15], [16]. Recently, four novel sex determining genes in the teleost fish species were identified, and they were *amhy* in the Patagonian pejerrey (*Odontesthes hatcheri*), *Amhr2* in fugu (*Takifugu rubripes*), *sdY* in rainbow trout (*Oncorhynchus mykiss*) and *Gsdf* in *Oryzias luzonensis* (a relative of medaka) [17], [18], [19], [20]. Together, sex determination and differentiation in fish are complex because of diverse range of species and common molecular mechanisms need to be studied.

Some heat shock proteins are closely related with gonad development and spermatogenesis. Down regulation of *Hsp10* will result in apoptosis in testis, which provided new aspects for understanding the mechanisms of germ cell apoptosis [21]. *Hspa2* (also named *Hsp70-2*) is a heat shock protein involved in maintenance of the nucleolus and centrosome integrity in cancer cells subjected to heat shock and protecting cells against cytotoxic stress [22], [23], [24], [25]. In addition, *Hspa2* plays an important role during meiosis in mouse [26], [27], even post-meiosis [28]. And male mice lacking *Hspa2* were infertile while females fertile [26]. *HSPA2* has a similar expression pattern in the male germ cells of human and mouse, and is also associated with sperm morphology and concentration [29], [30], [31], [32]. We have previously identified *Hsp10* in the swamp eel and found that down regulation of *Hsp10* is consistent with high apoptosis during the gonadal transformation. However, we are still lack of understanding of the roles and mechanisms of heat shock proteins in sexual differentiation.

In this study, we have cloned a testis-enriched *Hsp70* member, and further identified all members of *Hsp70* family in the swamp eel (*Monopterus albus*), a freshwater fish with a characteristic of natural sex reversal [33]. These results in the *Hsp70* family genes might shed light on *Hsp70* family evolution and their roles in gonadal development.

## Materials and Methods

### Animals and Reagents

The swamp eels were obtained from markets in the Wuhan area of China. Their gonads were confirmed by microscopic analysis of sections. Mice (Kunmingbai) were purchased from Wuhan Center for Disease Prevention and Control of Hubei in China. The polyclonal antibodies: anti-human NF-kB1 p50 (NLS) and anti-β-actin were purchased from Santa Cruz Biotechnology (CA, USA); the secondary antibody conjugated with AP was purchased from Vector (Buringame, CA, USA). The animals were treated in accordance with the guiding principles for biomedical research involving animals of Ethics and Animal Welfare Committee of College of Life Sciences of Wuhan University and the committee.

### Western Blot Analysis

Western blots were performed as the routine protocols [21]. We extracted proteins from freshly obtained tissues with buffer containing 50 mM Tris-Cl (pH 7.5), 140 mM NaCl, 1% Nonidet P-40, 2mM EDTA and complete inhibitor cocktail. Then we analyzed whole extract by Glycine-SDS-PAGE and transferred them onto 0.45 µm PVDF membrane (Hybond-P, Amersham Pharmacia Biotech, Sweden). The membranes were blocked with 5% low fat milk powder in TBST (20mM Tris-HCL pH7.5, 150mM NaCl, 0.1% Tween 20) and incubated with primary antibody at 4°C over night, and then with AP labeled secondary antibody for 1 hour at 25°C. The immunoreactive signals were revealed by NBT/BCIP regents.

### Immunoprecipitation (IP) and Mass Spectrometry (MS) Analysis

Proteins from the swamp eel and mouse gonads were extracted in IP buffer containing 50 mM Tris-Cl (pH 7.5), 140 mM NaCl, 1% Nonidet P-40, 2mM EDTA and complete inhibitor cocktail. The lysates were centrifuged at 10,000 g for 30 min at 4°C and precleared with Protein G PLUS-Agarose (Santa Cruz Biotech, USA). The precleared lysates (1 mg of protein) were incubated with 1 µg of anti-NF-kB1 p50 (NLS) antibody for 12 hrs at 4°C. Samples were washed three times with NP-40 lysis buffer, resuspended in 5X protein sample buffer, and placed for 5 min in boiling water, then placed in ice at once. A part of samples were analyzed by Western blot analysis with anti-NF-kB1 p50 (NLS) antibody. Most of samples from IP were analyzed by SDS-PAGE, and stained by Coomassie brilliant blue. Specific bands were cut from gels and put into 1.5mL EP tubes for MS analysis. The gels were washed twice with Milli-Q water for 15 min, and then washed three times in 25 mM NH_4_HCO_3_ and 50% CH_3_CN for 30 min with vortexing. The gels were then dehydrated in 100% CH_3_CN for 10 min with vortexing and allowed to air-dry for 1 h. Suspension of 1.5 mM trypsin (Promega, Madison, WI, USA) in 25 mM NH_4_HCO_3_ was added to gels, then digested at 37°C overnight. The 1.5ml EP tubes were then gently centrifuged, and the supernatant was removed for the MS analysis. Peptide mixtures were analyzed by the MALDI-TOF/TOF MS (Bruker-Daltonics AutoFlex TOF-TOF LIFT Mass Spectrometer, Bruker-Daltonics). Proteins were identified using the Mascot software (http://www.matrixscience.com) and SWISS-PROT, NCBInr database. Valid identification required a protein score greater than 65 when peptide mixtures were analyzed using the MALDI-TOF/TOF MS.

### Cloning of *Hsp70* Family Genes

Based on the amino acid sequences from MS analysis, the transcriptome data (unpublished) of the swamp eel were searched using BLASTp to find all related genes. PCR primers were designed to clone these genes. Primer sequences and amplification conditions were shown in Table 1. PCR was performed in a 20 µl reaction mixture containing 100ng DNA, 10mM Tris-HCl pH 8.3, 1.5mM MgCl_2_, 50mM KCl, 200 µM dNTP, 0.2 µM each primer, and 1 U Taq DNA polymerase. Amplified products were electrophoresed in a 0.8% agarose gel. The PCR products were cloned into T-vector and sequenced.

**Table 1:** The primers for sequencing.

Primer name	Primer sequences (5′-3′)	PCR
Hspa1a		
Hspa1a(F1)	ATGTCTGCAGCTAAAGGTGT	94°C, 30 s; 55°C, 30 s; 72°C, 2 min; 35cycles
Hspa1a(R1)	TCAATCTACCTCCTCAATAG	
Hspa1b		
Hspa1b(F1)	ATGTCCTCAGCTAAAGGAAT	94°C, 30 s; 59°C, 30 s; 72°C, 2 min; 35cycles
Hspa1b(R1)	TTAGTCCACTTCCTCAATAG	
Hspa4a		
Hspa4a(F1)	ATGGCAGTTGTCGGATTTGA	94°C, 30 s; 59°C, 30 s; 72°C, 2 min; 35cycles
Hspa4a(R1)	TTAATCAAGGTCCATGTCAG	
Hspa4b		
Hspa4b(F1)	ATGTCAGTGGTGGGATTTGA	94°C, 30 s; 59°C, 30 s; 72°C, 2 min; 35cycles
Hspa4b(R1)	TTAGTCAATGTTCATTTCAG	
Hspa4L		
Hspa4L(F1)	ATGTCAGTGGTAGGCATTGA	94°C, 30 s; 59°C, 30 s; 72°C, 2 min; 35cycles
Hspa4L(R1)	CTCCATCTCTTTCGTGCCAG	
Hspa5		
Hspa5(F1)	ATGAAGCTGTTATGGGTTGT	94°C, 30 s; 59°C, 30 s; 72°C, 2 min; 35cycles
Hspa5(R1)	CTACAACTCATCCTTCTCAT	
Hspa8a1		
Hspa8a1(F1)	ATGTCTAAAGGACCAGCAG	94°C, 30 s; 59°C, 30 s; 72°C, 2 min; 35cycles
Hspa8a1(R1)	TTAGTCGACCTCTTCAATGG	
Hspa8a2		
Hspa8a2(F1)	ATGTCAGTGGTAGGCATTGA	94°C, 30 s; 57°C, 30 s; 72°C, 2 min; 35cycles
Hspa8a2(R1)	TCAGTCGACTTCCTCGATGG	
Hspa8b1		
Hspa8b1(F1)	ATGTCCAAGGGACCAGCAGT	94°C, 30 s; 61°C, 30 s; 72°C, 2 min; 35cycles
Hspa8b1(R1)	TTAGTCAACCTCCTCAATGG	
Hspa8b2		
Hspa8b2(F1)	ATGTCTAAAGGACCAGCAGT	94°C, 30 s; 57°C, 30 s; 72°C, 2 min; 35cycles
Hspa8b2(R1)	CTGGTATAGCTTGGAGATGA	
Hspa9		
Hspa9(F1)	GGTTTCCAGCCAGATGTTCT	94°C, 30 s; 57°C, 30 s; 72°C, 2 min; 35cycles
Hspa9(R1)	TTAATCGTCATGACATATTG	
Hspa12a		
Hspa12a(F1)	ATGGCTAACCCTTCTCCAGC	94°C, 30 s; 59°C, 30 s; 72°C, 2 min; 35cycles
Hspa12a(R1)	TTAATGGCTCAGGAAGTCAA	
Hspa12b		
Hspa12b(F1)	GACAGTCCATCAGCCCCGTC	94°C, 30 s; 59°C, 30 s; 72°C, 2 min; 35cycles
Hspa12b(R1)	TCAGTTAGACAGGAAGTCTA	
Hspa14		
Hspa14(F1)	ATGGCTGCGATTGGAGTCCA	94°C, 30 s; 61°C, 30 s; 72°C, 2 min; 35cycles
Hspa14(R1)	TTATGAAGCAGCCGCTATGG	

### Phylogenetic Analysis

We searched homologous *Hsp70* genes of other vertebrates, including human, mouse, rat (*Rattus norvegicus*), camel (*Camelus dromedarius*), cattle (*Bos taurus*), platypus (*Ornithorhynchus anatinus*), chicken (*Gallus gallus*), frog (*Xenopus laevis*), zebrafish (*Danio rerio*), medaka, pufferfish (*Tetraodon nigroviridis*), stickleback (*Gasterosteus aculeatus*), fugu (*Takifugu rubripes*) and tilapia (*Orepchromis niloticus*) in NCBI and Ensembl databases. *Hsp70* family members have many synonyms, including *Hspa1a* (other aliases: *Hsp70-1a, Hsp70I, Hsp70-1, Hsp72* and *Hspa1*), *Hspa1b* (other aliases*: Hsp70-1, Hsp68* and *Hsp70.1*), *Hspa1L* (other aliases: *Hsp70-1L, Hsp70T, Hsp70-hom* and *hum70t*), *Hspa2* (other aliases: *Hsp70-3* and *Hsp70-2*), *Hspa4* (other aliases: *Hsp70RY, APG-2, Hsph2* and *HS24/P52*), *Hspa4L* (other aliases: *Osp94* and *APG-1*), *Hspa5* (other aliases: *Bip, Grp78* and *Mif2*), *Hspa6* (other aliases: *Hsp70B*), *Hspa8* (other aliases: *Hsc54, Hsc71, Hsc70, Hsp71, Hsp73, Hspa10, Lap1* and *Nip71*), *Hspa9* (other aliases: *CSA, Grp-75, Hspa9b, Grp75, Mot, Mot2, mtHsp75* and *Pbp74*), *Hspa12* and *Hspa14* (other aliases: *Hsp70-4* and *Hsp70L1*). We constructed phylogenetic tree using the Maximum Likelihood method in 100 bootstrap replicates (PHYLIP, version 3.68) (protein ID showed in Table S1).

### Sequence and Domain Analysis of Hsp70 Proteins

Hsp70 protein sequences and domains of the swamp eel were analyzed using Interproscan software. The protein sequences were aligned with the mammalian Hsp70 proteins. A complete protein alignment was generated by ClustalX V2.0 and Genedoc 2.7.0 (protein ID in Table S1), and the positives and identities were analyzed by Vector NT 12.0.

### RT-PCR Analysis

Total RNAs were isolated from adult tissues with TRIzol reagent (Invitrogen, Carlsbad, CA, USA) according to the manufacturer’s instructions. The cDNAs were reverse transcribed from the RNAs using the MMLV system (Promega, Madison, WI, USA) with 0.5 µg of oligo (dT)_18_ and 5 µg of total RNAs in a 25 µl reaction. PCR was performed in a 20 µl reaction mixture containing 100ng DNA, 10mM Tris-HCl pH 8.3, 1.5mM MgCl_2_, 50mM KCl, 200 µM dNTP, 0.2 µM each primer, and 1 U Taq DNA polymerase, and cDNA templates from testis, ovotestis and ovary of the swamp eel. Primers designed for semi-quantity PCR and amplification conditions were showed in Table 2. Amplified products were electrophoresed in a 2% agarose gel.

**Table 2:** Primers for semi-quantitative RT-PCR analysis.

Primer name	Primer sequences(5′–3′)	PCR
Hspa1a		
Hspa1a(F2)	TCCCAGCGACAGGCGACTAA	94°C, 30 s; 58°C, 30 s; 72°C, 30 s; 30cycles
Hspa1a(R2)	CTGGCTGATGTCCTTCTTGTGC	
Hspa1b		
Hspa1b(F2)	ACGAAATTGTCCTGGTTGGC	94°C, 30 s; 58°C, 30 s; 72°C, 30 s; 30cycles
Hspa1b(R2)	GGTATTGCGTTTGATTAGTGGTGT	
Hspa4a		
Hspa4a(F2)	CAGTTGAAATAGTGGGTGGAG	94°C, 30 s; 58°C, 30 s; 72°C, 30 s; 30cycles
Hspa4a(R2)	AGGAGATGGGATAAGGAA	
Hspa4b		
Hspa4b(F2)	CAGTGACTTTAGCGTATGG	94°C, 30 s; 58°C, 30 s; 72°C, 30 s; 30cycles
Hspa4b(R2)	CTCGCAGAAGTGGTTGA	
Hspa4L		
Hspa4L(F2)	TCCCTTTCCCATTACTCTTCG	94°C, 30 s; 58°C, 30 s; 72°C, 30 s; 30cycles
Hspa4L(R2)	CACGCACTTTGACCTTCACTTT	
Hspa5		
Hspa5(F2)	ACCAGCCTACTGTCACTATTA	94°C, 30 s; 58°C, 30 s; 72°C, 30 s; 30cycles
Hspa5(R2)	TTGTTCTTGTTGCCTGTG	
Hspa8a1		
Hspa8a1(F2)	ATGGATAAAGGGCAGATTC	94°C, 30 s; 58°C, 30 s; 72°C, 30 s; 30cycles
Hspa8a1(R2)	CCAGGGACAGAGGAGTGA	
Hspa8a2		
Hspa8a2(F2)	ACTCCTCTGTCCCTGGGTATTG	94°C, 30 s; 58°C, 30 s; 72°C, 30 s; 30cycles
Hspa8a2(R2)	CCTTGCTGAGACGACCCTTG	
Hspa8b1		
Hspa8b1(F2)	GGGGTGCTCATTCAGGTGTT	94°C, 30 s; 58°C, 30 s; 72°C, 30 s; 30cycles
Hspa8b1(R2)	TCTGCTTCCTGAACCATACGC	
Hspa8b2		
Hspa8b2(F2)	GGAGGCTGAGCAAGGAGGAA	94°C, 30 s; 58°C, 30 s; 72°C, 30 s; 30cycles
Hspa8b2(R2)	CTGTCCAGCCAGGCAATAACT	
Hspa9		
Hspa9(F2)	TGTGGGCATACCTGCTAAACG	94°C, 30 s; 58°C, 30 s; 72°C, 30 s; 30cycles
Hspa9(R2)	TTGGTAGCCTGAGAATCAAAGAAA	
Hspa12a		
Hspa12a(F2)	CTCCAACCACAATCCTGCTGAC	94°C, 30 s; 58°C, 30 s; 72°C, 30 s; 30cycles
Hspa12a(R2)	GGCTTTGACTCGCTTCCCATT	
Hspa12b		
Hspa12b(F2)	GCAGCCTGGGTGGACCTAAC	94°C, 30 s; 58°C, 30 s; 72°C, 30 s; 30cycles
Hspa12b(R2)	TGGTGATGGTGGGTTGGAAGA	
Hspa14		
Hspa14(F2)	TGAGCGTGACAGTGCTACAGG	94°C, 30 s; 58°C, 30 s; 72°C, 30 s; 30cycles
Hspa14(R2)	TCAAAGTCAATGCCGTCGTG	
Hprt		
Hprt(F)	GAACAGTGACCGCTCCATCC	94°C, 30 s; 58°C, 30 s; 72°C, 30 s; 30cycles
Hprt(R)	TCTTCATCGTCTTTCCCGTGTC	

## Results

### Identification of a Testis-enriched *Hsp70* Gene in the Swamp Eel

**Figure 1:**
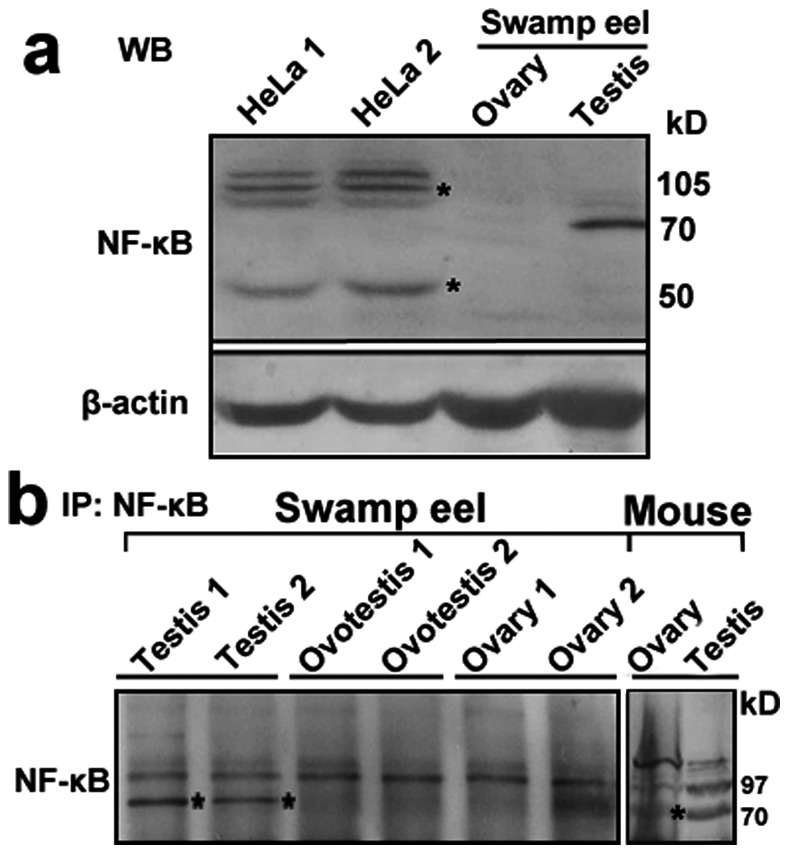
Western blot analysis of unknown testis-enriched protein in mouse and swamp eel. **a.** Western blot analysis using anti-NF-kB1 antibody in HeLa cells and the swamp eels showed two bands: 50kD (P50) and 105kD (P105) (stars denote the bands) in HeLa cells (two repeat samples), while only a dominant 70 KD band in testis of the swamp eel. β-actin protein was used as an internal control. Molecular weight sizes for proteins were shown on the right. **b.** Immunoprecipitation using anti-NF-kB1 antibody enriched the unknown testis-enriched protein in the swamp eel and mouse. Testis samples showed more obvious band of 70 kD in the swamp eel and mouse. Stars denote the band.

Western blot analysis using anti-NF-kB1 antibody in HeLa cells showed normal two bands [50 kD (P50) and 105 kD (P105)], while only a testis-enriched band of 70 kD in the swamp eel was observed (Figure 1a). Further immunoprecipitation using anti-NF-kB1 antibody clearly showed the object unknown protein in testis samples of both the swamp eel and mouse (Figure 1b). The MALDI-TOF-TOF MS analysis was used to identify the proteins of both the swamp eel and mouse. The results indicated that the unknown testis-enriched protein was similar to *Hsp70* family member *Hspa2* or *Hspa8* (Table 3), which had a conserved domain Hspa1–2_6–8-like_NBD (nucleotide-binding domain).

**Table 3:** MS results of the unknown proteins from mouse and the swamp eel.

Species # Protein groups	Amino Acid Sequences	GenBank Accession No.	Identified Protein Names	Coverage	MASCOT Scores
**Mouse**					
#1–1	K.DAGTITGLNVLR.I	NP_001002012	Heat shock protein 2 (Hspa2) [*Mus musculus*]	17.54%	805.6
	K.LDKGQIQEIVLVGGSTR.I				
	K.LLQDFFNGK.E				
	K.NAVESYTYNIK.Q				
	K.NQVAMNPTNTIFDAK.R				
	K.VQSAVITVPAYFNDSQR.Q				
	R.IINEPTAAAIAYGLDK.G				
	R.IINEPTAAAIAYGLDKK.V				
	R.TTPSYVAFTDTER.L				
#2–1	K.DAGTIAGLNVLR.I	NP_112442	Heat shock cognate 71 (Hspa8) [*Mus musculus*]	10.68%	1339.8
	K.LLQDFFNGK.E				
	K.TVTNAVVTVPAYFNDSQR.Q				
	R.IINEPTAAAIAYGLDK.G				
	R.IINEPTAAAIAYGLDKK.V				
	R.TTPSYVAFTDTER.L				
#3–1	K.APQVSTPTLVEAAR.N	NP_033784	Serum albumin precursor [*Mus musculus*]	6.91%	690.1
#4–1	R.NHTLQKWHDK.T	XP_993489.1	similar to Protein AATF [*Mus musculus*]	1.88%	453.8
**Swamp eel**					
#1–1	K.TVLTQEALISVK.G	XP_003976390.1	PREDICTED: protein slowmo homolog 2-like [*Takifugu rubripes*]	6.35%	963
#2–1	K.YKLIKLGMSK.V	CAG00227.1	unnamed protein product [*Tetraodon nigroviridis*]	2.89%	849.6
#3–1	K.FTASGGEGMLSILKK.S	CAG11484.1	unnamed protein product [*Tetraodon nigroviridis*]	2.15%	374
#4–1	K.HQRELENLQEEKER.L	CAF91893.1	unnamed protein product [*Tetraodon nigroviridis*]	0.8%	897.9
#5–1	K.LENVLLDENLNIK.I	XP_003444058.1	PREDICTED: NUAK family SNF1-like kinase 1 [*Oreochromis niloticus*]	2.02%	357.1
#6–1	K.TVNNAVITVPAYFNDSQR.Q	NP_001098270.1	Heat shock cognate 71 (Hspa8) [*Oryzias latipes*]	2.62%	185.5
#7–1	R.ISITLVSVPLIVR.Y	XP_003973504.1	PREDICTED: protein FAM210B-like [*Takifugu rubripes*]	4.44%	483.8

**Figure 2:**
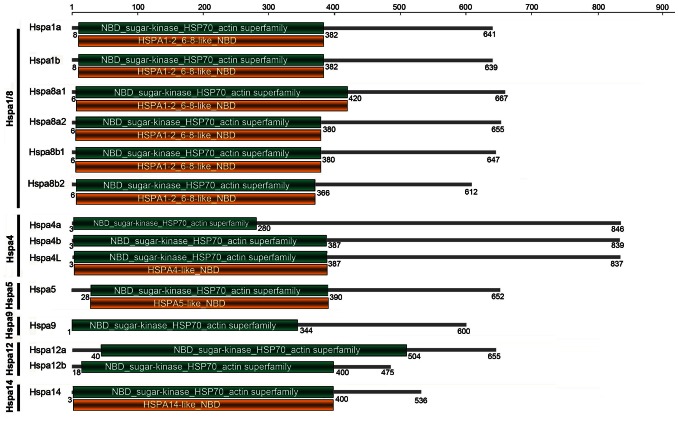
Conserved domain analysis of Hsp70 family members in swamp eel. All the 14 proteins had NBD_sugar-kinase_HSP70_actin superfamily domain (green bars), and some genes had their own conserved domain (yellow bars). Hspa1a/b and Hspa8a1/a2/b1/b2 had a common domain (HSPA1–2_6–8-like_NBD). The numbers on the bars denote amino acid positions. GenBank accession numbers were shown in Table S1.

To further characterize the testis-enriched gene, we cloned 14 members of *Hsp70* family from the swamp eel (Table S1 and Figure 2). Conserved domain analysis showed that *Hspa1a/b* and *Hspa8a/b* had the same Hspa1–2_6–8-like_NBD domain (Figure 2). Further sequence analysis showed that there were four copies of *Hspa8 (–8a1/−8a2 and –8b1/−8b2)*, three copies of *Hspa4 (–4a/−4b and –4L)*, two copies of *Hspa1 (–1a/−1b)* and *Hspa12 (–12a/−12b)*, one copy of *Hspa5, Hspa9* and *Hspa14* in the swamp eel.

### Phylogenetic and Copy Number Analysis of *Hsp70* Family Genes in Vertebrates

**Figure 3:**
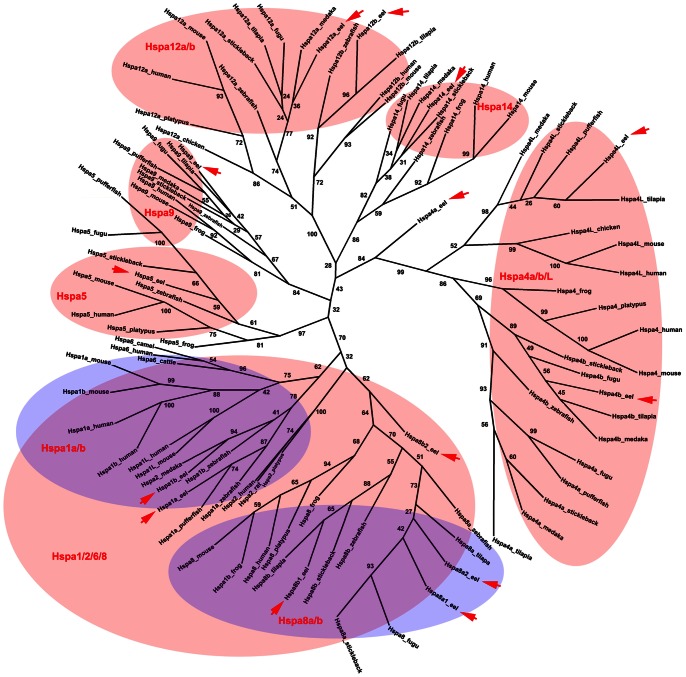
Phylogenetic tree of Hsp70 family in vertebrates. The phylogenetic tree was constructed using the Maximum Likelihood method based on the amino acids of Hsp70 members from human, mouse, rat, camel, cattle, platypus, chicken, frog, zebrafish, pufferfish, fugu, stickleback, medaka, tilapia and the swamp eel. The protein sequences were from NCBI and Ensembl database (protein ID in Table S1). 14 proteins of the swamp eel had been grouped into 7 cluster of Hsp70 family (red arrows). The numbers at the nodes indicate bootstrap values.

**Figure 4:**
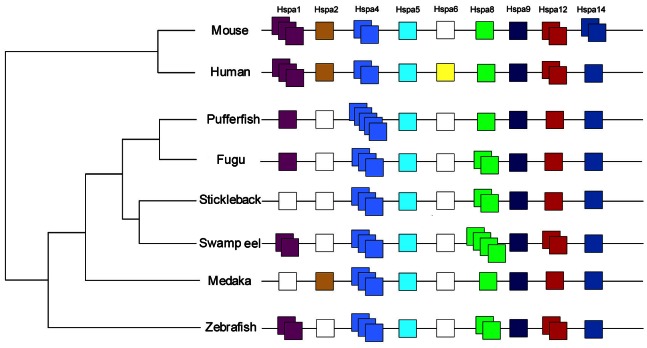
Copy number variation analysis of *Hsp70* family genes from various vertebrates. *Hspa5* and *Hspa9* had one copy in these species, while copy numbers of the other genes ranged from 0 to 5. *Hspa4* and *Hspa8* had duplicated in fishes, while *Hspa2* and *Hspa6* may be lost in fishes (previously named *Hspa2* in medaka would be *Hspa1*). Squares with different colors represent different genes and copies, while white squares denote the genes, which have not been detected in these species.

As *Hsp70* family members have many synonyms, we aligned all *Hsp70* members of vertebrates and analyzed their clusters using the Maximum Likelihood method. A phylogenetic tree was finally constructed (Figure 3). Each *Hsp70* gene in the swamp eel could be grouped into a cluster of *Hsp70* family. The evolutionary analysis of these *Hsp70* family genes in vertebrates showed that more copies of *Hsp70* members were observed in fishes than mammals (Figure 4), especially 3–5 copies of *Hspa4* in the fish species. Because of a high level of homology between *Hspa8* and *Hspa2*, we further compared their sequence homology to determine the branching of the *Hspa8* and *Hspa2* genes. The protein sequence alignments of the swamp eel Hspa8 (–8a1/−8a2 and –8b1/−8b2) with the mammalian Hspa8 and Hspa2 indicated that the swamp eel Hspa8b2 was more similar to Hspa8 (positive and identity of Hspa8b2 with Hspa8 of human and mouse: 100% and 86.7%; Hspa8b2 with Hspa2 of human and rat: 98.9% and 82.5%, respectively) (Figure S1 a, b, c). Thus four copies of *Hspa8* genes (named as *Hspa8a1/−a2* and *Hspa8b1/−b2*) in the swamp eel were further confirmed. *Hspa2* and *Hspa6* may be lost in the teleost fish species (Figure 4), and previously named *Hspa2* in medaka may be *Hspa1b* (Figure 3).

### Expression Analysis of *Hsp70* Family Genes

**Figure 5:**
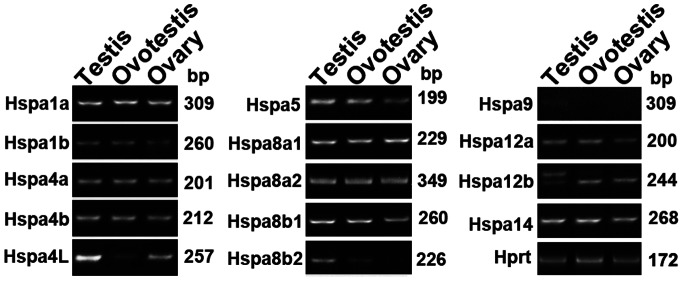
RT-PCR analysis of *Hsp70* gene expression in gonad samples of the swamp eel. Most of the *Hsp70* family genes were expressed equally in different types of gonads except *Hspa4L, Hspa5, Hspa8b2, Hspa9* and *Hspa12a*. *Hspa8b2* was expressed differentially among testis, ovotestis and ovary. *Hprt* was used as an internal control. Length sizes of amplified products were shown on the right.

We further analyzed expression pattern of *Hsp70* family genes in three types of gonads in the swamp eel by RT-PCR. Most of the *Hsp70* genes were expressed equally in different types of gonads except *Hspa4L, Hspa5, Hspa8b2* and *Hspa9. Hspa9* was expressed faintly in gonads. Only *Hspa8b2* and *Hspa5* were upregulated from ovary, ovotestis to testis (Figure 5). *Hspa8b2* gene has Hspa1–2_6–8-like_NBD conserved domain, which is similar to the protein identified by the MS analysis. Together with phylogenetic analysis, these results indicated that the testis-enriched gene may be *Hspa8b2* in the swamp eel, instead of *Hspa2*.

### *Hspa8* is Evolutionarily Conserved with Three Conserved Domains

**Figure 6:**
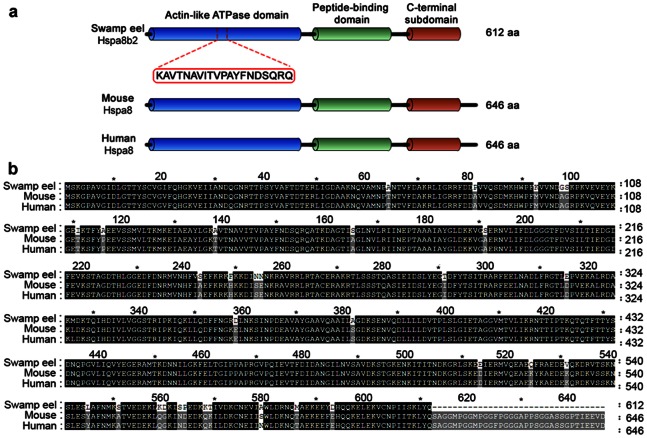
Sequence analysis of Hspa8b2 protein of the swamp eel. **a.** Cartoon showed a linear representation of the swamp eel Hspa8b2 in comparison with Hspa8 of mouse and human. Different colors showed three conserved domains. Protein sequence in red square showed the corresponding sequence, which is similar to Hspa8 identified by MS analysis. **b.** A complete protein alignment of the swamp eel Hspa8b2 with Hspa8 of mouse and human using ClustalX V2.0 and Genedoc 2.7.0 (protein ID in Table S1), and the positive and identity of the alignments were 100% and 86.7% respectively. White letters on black background indicated identical amino acids.

Protein sequence of Hspa8b2 in the swamp eel was further aligned with the mammalian Hspa8, which showed a high level of sequence homology. Conserved domains were further analyzed using Interproscan software. Three conserved domains were identified, which were actin-like ATPase domain, peptide-binding domain and C-terminal subdomain (Figure 6a). In addition, in comparison with those of mouse and human, the protein size of the swamp eel Hspa8b2 was shorted of 34 amino acids, and the positive and identity of the alignments were 100% and 86.7% respectively (Figure 6a, b).

## Discussion

*Hsp70* family genes play crucial roles in protecting cells against heat and other stresses in most animal species. In the present study, we have identified 14 members of *Hsp70* family in the swamp eel. In comparison with this, there are about 10 members in the *Hsp70* family in other fish species including zebrafish, medaka, pufferfish, threespine stickleback, pufferfish and fugu. Particularly *Hspa8* has expended to 4 members in the swamp eel genome, while other fishes have 1–2 copies. The expansion of *Hsp70* family members in the swamp eel may reflect its adaptation to physiological change in sex reversal and water/air-breathing of the species. The expansion to 88 members of the *Hsp70* family in the Pacific oyster *Crassostrea gigas* genome is a typical example to adapt to sessile life in the highly stressful intertidal zone [34].

As a multigene family, *Hsp70* genes have evolved by duplications, subfunctionalization, nonfunctionalization or even loss. The birth-and-death model may explain well the evolution mechanism of the *Hsp70* family. New genes are created by gene duplication and some duplicated genes stay in the genome for a long time, whereas others are inactivated or deleted from the genome [35], [36]. Gene duplication is one of the main forces acting on the evolution of organisms, which creates new genes for adaptation evolution in favor of natural selection.

*Hsp70* genes have many functions including protein transportation between cellular compartments, protein folding, degradation of unstable and misfolded proteins, and control of regulatory proteins [37], [38], [39]. Some Hsp70 family proteins can prevent caspase-independent cell death [40], [41], while most *Hsp70* genes play crucial roles in protecting cells against heat and other stresses [34]. *Hsp70* family members in cells have different but closely related gene products: the stress-inducible form such as *Hspa1a, Hspa1b* and *Hspa6*, and the constitutively expressed form, such as *Hspa1L, Hspa2, Hspa5, Hspa8* and *Hspa9* [42]. Some *Hsp70* family members have tissue-special functions. For example, Hspa1a/b proteins can be released from cells and act as messengers and play a role in the immune system [43], [44]. Hspa1L and Hspa2 are sperm proteins which are important for sperm functions [45], [46]. *Hspa8* is important in viral assembly in cells, independently of its chaperone function [47]. Some heat shock proteins have functions in gonad development, for example, Hsp10 and Hspa2 [21], [30]. Here, we have further identified testis-enriched *Hspa8b2* in the swamp eel. Phylogenetic analysis, sequence characteristic and the expression pattern support this gene as a candidate for gonad development/spermatogenesis. This finding derives from Western blot analysis of gonadal samples using NF-kB1 antibody. In fact, it has been predicted that NF-kB protein could interact selectively and non-covalently with heat shock protein 70, which was based on Gene Ontology (Function GO:0031072) [48]. Further studies of roles and functions of *Hspa8b2* will provide comprehensive understanding of the molecular mechanisms of gonadal transformation from ovary to testis via ovotestis in the special fish species, the swamp eel.

## Supporting Information

Figure S1 **Protein alignments of the swamp eel Hspa8b2 with Hspa2 and Hspa8 of mammals.** **a.** Complete protein alignments of Hspa8a1/a2/b1/b2 of the swamp eel with Hspa8 of human and mouse. **b.** Complete protein alignments of Hspa8a1/a2/b1/b2 of the swamp eel with Hspa2 of human and rat. **c.** Complete protein alignments of Hspa8b2 of the swamp eel with Hspa2 of human and rat. White letters on black background indicated identical amino acids, and these genes accession numbers were showed in Table S1.
(TIF)

Table S1 **List of protein sequences used for sequence analysis.**
(DOC)
