# Supplementary figures and images for: Identification of a Testis-Enriched Heat Shock Protein and Fourteen Members of Hsp70 Family in the Swamp Eel
Source: PLoS One. 2013 Jun 4;8(6):e65269. doi: 10.1371/journal.pone.0065269 (PMC3672149; doi:10.1371/journal.pone.0065269)

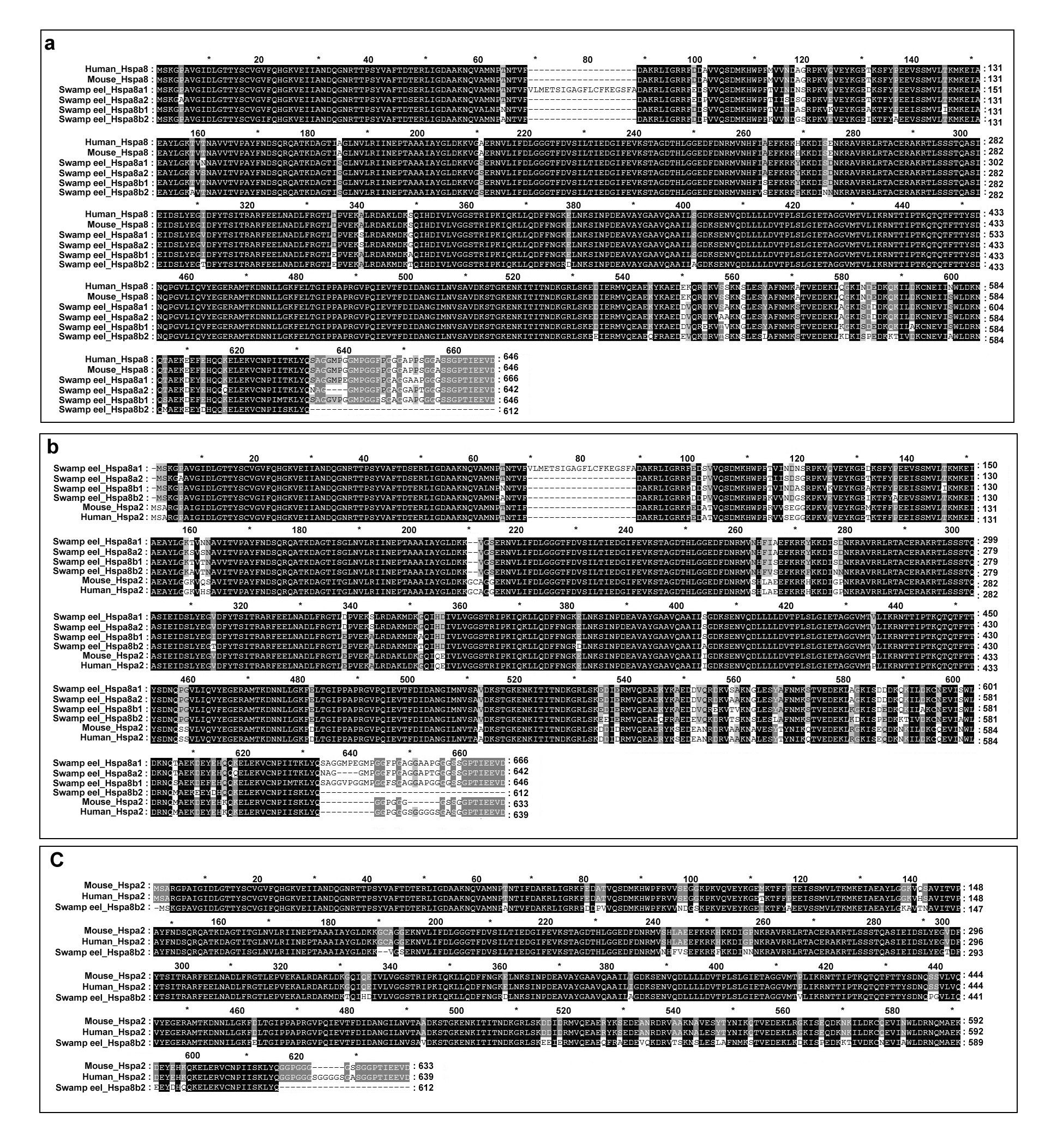

Supplement: Figure S1 — Protein alignments of the swamp eel Hspa8b2 with Hspa2 and Hspa8 of mammals. a. Complete protein alignments of Hspa8a1/a2/b1/b2 of the swamp eel with Hspa8 of human and mouse. b. Complete protein alignments of Hspa8a1/a2/b1/b2 of the swamp eel with Hspa2 of human and rat. c. Complete protein alignments of Hspa8b2 of the swamp eel with Hspa2 of human and rat. White letters on black background indicated identical amino acids, and these genes accession numbers were showed in Table S1. (TIF) [file pone.0065269.s001.tif]
