# Supplementary material for: Identification of a Testis-Enriched Heat Shock Protein and Fourteen Members of Hsp70 Family in the Swamp Eel
Source: PLoS One. 2013 Jun 4;8(6):e65269. doi: 10.1371/journal.pone.0065269 (PMC3672149; doi:10.1371/journal.pone.0065269)
Supplement: Table S1 — List of protein sequences used for sequence analysis. (DOC) [file pone.0065269.s002.doc]

Table S1. List of protein sequences used for sequence analysis.

| **Protein ID** | **Gene name** | **Resource** | **Protein ID** | **Gene name** | **Resource** |
| --- | --- | --- | --- | --- | --- |
| ENSTNIG00000004281 | Hspa1a_pufferfish | *Tetraodon nigroviridis* | NP_001098270.1 | Hspa8_medaka | *Oryzias latipes* |
| ENSDARG00000029688 | Hspa1a_zebrafish | *Danio rerio* | ENSONIG00000004759 | Hspa8a_tilapa | *Oreochromis niloticus* |
| AAH09322.1 | Hspa1a_human | *Homo sapiens* | ENSGACG00000020117 | Hspa8a_stickleback | *Gasterosteus aculeatus* |
| NP_034609.2 | Hspa1a_mouse | *Mus musculus* | ENSDARG00000068992 | Hspa8a_zebrafish | *Danio rerio* |
| NP_001167480.1 | Hspa1b_frog | *Xenopus laevis* | ENSONIG00000011144 | Hspa8b_tilapia | *Oreochromis niloticus* |
| ENSDARG00000056210 | Hspa1b_zebrafish | *Danio rerio* | ENSGACG00000010517 | Hspa8b_stickleback | *Gasterosteus aculeatus* |
| EAX03529.1 | Hspa1b_human | *Homo sapiens* | ENSDARG00000037403 | Hspa8b_zebrafish | *Danio rerio* |
| AAI51108.1 | Hspa1b_mouse | *Mus musculus* | ENSTRUG00000007906 | Hspa9_fugu | *Takifugu rubripes* |
| AAH34483.1 | Hspa1L_human | *Homo sapiens* | ENSORLG00000010637 | Hspa9_medaka | *Oryzias latipes* |
| AAI29896.1 | Hspa1L_mouse | *Mus musculus* | ENSGACG00000019542 | Hspa9_stickleback | *Gasterosteus aculeatus* |
| ENSOANG00000009457 | Hspa2_platypus | *Ornithorhynchus anatinus* | ENSONIG00000004081 | Hspa9_tilapia | *Oreochromis niloticus* |
| ENSORLG00000006132 | Hspa2_medaka | *Oryzias latipes* | ENSTNIG00000005994 | Hspa9_pufferfish | *Tetraodon nigroviridis* |
| AAH81803.1 | Hspa2_rat | *Rattus norvegicus* | ENSDARG00000003035 | Hspa9_zebrafish | *Danio rerio* |
| AAH36107.1 | Hspa2_human | *Homo sapiens* | AAH57343.1 | Hspa9_mouse | *Mus musculus* |
| ENSOANG00000007430 | Hspa4_platypus | *Ornithorhynchus anatinus* | NP_001079627.1 | Hspa9_frog | *Xenopus laevis* |
| NP_001083317.1 | Hspa4_frog | *Xenopus laevis* | NP_004125.3 | Hspa9_human | *Homo sapiens* |
| NP_002145.3 | Hspa4_human | *Homo sapiens* | ENSOANG00000014135 | Hspa12a_platypus | *Ornithorhynchus anatinus* |
| AAH03770.1 | Hspa4_mouse | *Mus musculus* | ENSTRUG00000004009 | Hspa12a_fugu | *Takifugu rubripes* |
| ENSGACG00000018188 | Hspa4a_stickleback | *Gasterosteus aculeatus* | ENSGACG00000014625 | Hspa12a_stickleback | *Gasterosteus aculeatus* |
| ENSTNIG00000008942 | Hspa4a_pufferfish | *Tetraodon nigroviridis* | ENSTNIG00000004924 | Hspa12a_tilapia | *Oreochromis niloticus* |
| ENSORLG00000005958 | Hspa4a_medaka | *Oryzias latipes* | ENSDARG00000070603 | Hspa12a_zebrafish | *Danio rerio* |
| ENSTRUG00000006546 | Hspa4a_fugu | *Takifugu rubripes* | ENSORLG00000001174 | Hspa12a_medaka | *Oryzias latipes* |
| ENSONIG00000012895 | Hspa4a_tilapia | *Oreochromis niloticus* | NP_079291.2 | Hspa12a_human | *Homo sapiens* |
| ENSGACG00000020732 | Hspa4b_stickleback | *Gasterosteus aculeatus* | XP_421779.3 | Hspa12a_chicken | *Gallus gallus* |
| ENSDARG00000018989 | Hspa4b_zebrafish | *Danio rerio* | NP_780408.1 | Hspa12a_mouse | *Mus musculus* |
| ENSORLG00000001448 | Hspa4b_medaka | *Oryzias latipes* | NP_001036151.1 | Hspa12b_zebrafish | *Danio rerio* |
| ENSTRUG00000008366 | Hspa4b_fugu | *Takifugu rubripes* | XP_003452414.1 | Hspa12b_tilapia | *Oreochromis niloticus* |
| ENSONIG00000008454 | Hspa4b_tilapia | *Oreochromis niloticus* | NP_082582.1 | Hspa12b_mouse | *Mus musculus* |
| NP_001012594.1 | Hspa4L_chicken | *Gallus gallus* | NP_443202.3 | Hspa12b_human | *Homo sapiens* |
| NP_035150.3 | Hspa4L_mouse | *Mus musculus* | NP_056580.2 | Hspa14_mouse | *Mus musculus* |
| NP_055093.2 | Hspa4L_human | *Homo sapiens* | NP_001092168.1 | Hspa14_frog | *Xenopus laevis* |
| ENSGACG00000008210 | Hspa4L_stickleback | *Gasterosteus aculeatus* | NP_057383.2 | Hspa14_human | *Homo sapiens* |
| ENSTNIG00000017360 | Hspa4L_pufferfish | *Tetraodon nigroviridis* | ENSTRUG00000012564 | Hspa14_fugu | *Takifugu rubripes* |
| ENSONIG00000020135 | Hspa4L_tilapia | *Oreochromis niloticus* | ENSORLG00000012603 | Hspa14_medaka | *Oryzias latipes* |
| ENSORLG00000015961 | Hspa4L_medaka | *Oryzias latipes* | ENSGACG00000019293 | Hspa14_stickleback | *Gasterosteus aculeatus* |
| ENSGACG00000016633 | Hspa5_stickleback | *Gasterosteus aculeatus* | ENSONIG00000014177 | Hspa14_tilapia | *Oreochromis niloticus* |
| ENSTNIG00000018747 | Hspa5_pufferfish | *Tetraodon nigroviridis* | ENSDARG00000058030 | Hspa14_zebrafish | *Danio rerio* |
| ENSDARG00000004665 | Hspa5_zebrafish | *Danio rerio* | KC455493 | Hspa1a_eel | *Monopterus albus* |
| ENSOANG00000013291 | Hspa5_platypus | *Ornithorhynchus anatinus* | KC455494 | Hspa1b_eel | *Monopterus albus* |
| ENSTRUG00000003346 | Hspa5_fugu | *Takifugu rubripes* | KC455495 | Hspa4a_eel | *Monopterus albus* |
| AAH50927.1 | Hspa5_mouse | *Mus musculus* | KC455496 | Hspa4b_eel | *Monopterus albus* |
| AAI12964.1 | Hspa5_human | *Homo sapiens* | KC455497 | Hspa4L_eel | *Monopterus albus* |
| AAH41200.1 | Hspa5_frog | *Xenopus laevis* | KC455498 | Hspa5_eel | *Monopterus albus* |
| NP_002146.2 | Hspa6_human | *Homo sapiens* | KC455499 | Hspa8a1_eel | *Monopterus albus* |
| ADO12067.1 | Hspa6_camel | *Camelus dromedarius* | KC455500 | Hspa8a2_eel | *Monopterus albus* |
| XP_002685896.1 | Hspa6_cattle | *Bos taurus* | KC455501 | Hspa8b1_eel | *Monopterus albus* |
| ENSOANG00000014580 | Hspa8_platypus | *Ornithorhynchus anatinus* | KC455502 | Hspa8b2_eel | *Monopterus albus* |
| ENSTRUG00000005265 | Hspa8_fugu | *Takifugu rubripes* | KC455503 | Hspa9_eel | *Monopterus albus* |
| NP_001079632.1 | Hspa8_frog | *Xenopus laevis* | KC455504 | Hspa12a_eel | *Monopterus albus* |
| AAH19816.1 | Hspa8_human | *Homo sapiens* | KC455505 | Hspa12b_eel | *Monopterus albus* |
| AAH94900.1 | Hspa8_mouse | *Mus musculus* | KC455506 | Hspa14_eel | *Monopterus albus* |
